# A pediatric case of citrin deficiency presenting with recurrent hypertriglyceridemic pancreatitis-a case report

**DOI:** 10.3389/fped.2026.1816553

**Published:** 2026-06-08

**Authors:** Ruyi Ye, Puhong Zhang, Yong Gu, Gang Feng, Yue Qu, Hongchuan Zhang, Wei Wei, Lizhu Huang

**Affiliations:** 1 Department of Pediatrics, The First Affiliated Hospital of Wannan Medical University (Yijishan Hospital of Wannan Medical University), Wuhu, China; 2 Department of Critical Care Medicine, The First Affiliated Hospital of Wannan Medical University (Yijishan Hospital of Wannan Medical University), Wuhu, China; 3 Department of Laboratory Medicine, The First Affiliated Hospital of Wannan Medical University (Yijishan Hospital of Wannan Medical University), Wuhu, China; 4 Department of Laboratory Medicine, Wannan Medical University, Wuhu, China; 5 Department of Radiology, The First Affiliated Hospital of Wannan Medical University (Yijishan Hospital of Wannan Medical University), Wuhu, China

**Keywords:** citrin deficiency, *SLC25A13* gene, failure to thrive and dyslipidemia caused by citrin deficiency, hypertriglyceridemia, hypertriglyceridemic pancreatitis

## Abstract

Citrin deficiency (CD) is a rare autosomal recessive metabolic disorder caused by pathogenic variants in the *SLC25A13* gene, which encodes the mitochondrial aspartate-glutamate carrier 2, also known as citrin. We describe an 11-year-old Chinese boy presenting with recurrent acute pancreatitis secondary to severe hypertriglyceridemia during the FTTDCD/post-NICCD stage of citrin deficiency. The patient was relatively thin (his height and weight were at the 10th percentile on the growth curve for Chinese children), had a strong preference for soy products and an aversion to carbohydrates. Laboratory tests at presentation revealed severe hypertriglyceridemia (28.96 mmol/L), with a previously documented peak of 30.35 mmol/L. Abdominal computed tomography showed diffuse pancreatic enlargement and peripancreatic inflammatory changes, which, together with compatible abdominal pain, supported the diagnosis of acute pancreatitis. Genetic sequencing identified compound heterozygous pathogenic mutations in the *SLC25A13* gene (exon 9: c.852_855delTATG; intron 6: c.615+5G > A). Plasma ammonia and citrulline levels were within normal limits. All of these findings supported a diagnosis of failure to thrive and dyslipidemia caused by citrin deficiency (FTTDCD) in the post-NICCD phase. Management involved plasma exchange, a high-protein/high-fat/low-carbohydrate diet, and medium-chain triglyceride (MCT) supplementation, leading to clinical improvement. However, poor dietary adherence during follow-up resulted in two readmissions for recurrent pancreatitis. This case highlights that citrin deficiency should be considered in children with recurrent acute pancreatitis associated with severe hypertriglyceridemia, especially when accompanied by carbohydrate aversion and unusual dietary preferences, even in the absence of hyperammonemia or hypercitrullinemia.

## Introduction

Acute pancreatitis is most commonly attributed to excessive alcohol intake or acute biliary obstruction. Hypertriglyceridemia represents only 5% of acute pancreatitis cases, typically occurring when triglyceride levels exceed 1,000 mg/dL (11.29 mmol/L) (1). Identifying the underlying etiology of hypertriglyceridemia is essential for guiding targeted therapeutic interventions. Here, we report a child with recurrent pancreatitis who presented with recurrent abdominal pain and serum triglyceride levels reaching as high as 28.96 mmol/L, ultimately confirmed to be caused by citrin deficiency.

The *SLC25A13* gene encodes a mitochondrial membrane protein named citrin, which is required to move electrons from cytosolic NADH to the mitochondrial matrix in hepatocytes (2, 3). Dysfunction of citrin leads to impaired urea cycle function, disruption of the malate-aspartate shuttle (MAS), and an imbalance in the NADH/NAD⁺ ratio, which collectively contribute to the clinical manifestations of citrin deficiency (CD) (2–4). Citrin deficiency has age-related phenotypes: neonatal intrahepatic cholestasis caused by CD (NICCD, OMIM #605814), failure to thrive and dyslipidemia caused by CD (FTTDCD), and type II citrullinemia (CTLN2, OMIM #603471), also referred to as adolescent and adult citrin deficiency (AACD) (2).

Although previous cases of CD-associated pancreatitis mainly occurred at the AACD stage accompanied by markedly elevated plasma ammonia and citrulline concentrations, serum triglyceride levels were not documented in those reports (5, 6). To our knowledge, this is among the first reported pediatric FTTDCD cases in which recurrent acute pancreatitis was driven predominantly by severe hypertriglyceridemia, highlighting the clinical complexity and diversity of the disease at this stage (Table 1). While previous reports of CD-associated pancreatitis have occurred predominantly in the hyperammonemia AACD stage, our case uniquely demonstrates that severe hypertriglyceridemia with recurrent pancreatitis can be the predominant presenting feature during the FTTDCD stage, even in the absence of elevated ammonia or citrulline.

**Table 1:** Comparison of clinical features between the present case and previously reported CD-associated pancreatitis cases.

Feature	Present case	Case1.Ikeda.et.al..2004.	Case2.Ikeda.et.al..2004.	Case3.Ikeda.et.al..2004.	Case4.Lai.et.al..2024.
Gender	Male	Male	Male	Male	Male
Age at pancreatitis (yrs)	9	24	14	33	17
Age at CD diagnosis (yrs)	11	33	25	40	18
Fasting blood glucose (mmol/L)	5.22	5.55	4.3	NR	NR
Plasma ammonia (*μ*mol/L)	40 (normal)	139 (↑)	98 (↑)	125 (↑)	Increased
Plasma citrulline (μmol/L)	26.5 (normal)	1,244.6 (↑↑)	794.6 (↑↑)	186.9 (↑)	175.6 (↑)
Plasma arginine (μmol/L)	11.7 (normal)	551.2 (↑↑)	119.7 (↑)	175.0 (↑)	26.6 (normal)
Serum amylase (U/L)	51–100 (normal)	104(normal)	89(normal)	748(↑↑)	239 -996(↑↑)
Hypertriglyceridemia	Yes (28.96 mmol/L↑↑)	NR	NR	NR	NR
Carbohydrate aversion	Yes	NR	NR	NR	NR
Neuropsychiatric symptoms	None	Encephalopathy	Encephalopathy	Encephalopathy	NR
Imaging or pathological findings	Abdominal CT: pancreatic swelling and peripancreatic exudation, no parenchymal necrosis, normal liver density	ERCP: marked main pancreatic duct dilatation with intraductal stones	Autopsy: small pancreas, severe fibrosis, diffuse acinar atrophy	Abdominal CT: atrophied pancreas with dotty calcification; histopathology: acinar atrophy, mild fibrosis, protein plugs in ducts	Abdominal CT: pancreatic swelling and peripancreatic exudation, no parenchymal necrosis, hepatomegaly with decreased liver density
CD stage	FTTDCD	AACD	AACD	AACD	AACD
*SLC25A13* gene mutation	c.852_855del/c.615+5G>A	c.815_819del/c.674C>A^a^	c.852_855del/c.1177+1G>A^a^	c.1177+1G>A^a^	c.1638_1660dup/c.852_855del
Outcome	Survival	Survival	Died	Died	Survival

CD, citrin deficiency; NR, not reported; ↑, elevated; ↑↑, markedly elevated; FTTDCD, failure to thrive and dyslipidemia caused by citrin deficiency; AACD, adolescent and adult citrin deficiency; CT, computed tomography; ERCP, endoscopic retrograde cholangiopancreatography.

Unit conversions were applied where applicable. reference ranges vary by laboratory; please refer to original publications for detailed reference range.

a c.674C>A (p.Ser225Ter), formerly S225X;c.1177+1G>A (p.340_392del), formerly IVS11+1G>A (9).

## Case presentation

An 11-year-old boy was admitted to our hospital with worsening recurrent abdominal pain for one day. Over the preceding year, he had experienced three similar episodes requiring hospitalization. During each acute pancreatitis episode, the clinical manifestations included acute abdominal pain (rated 6–9 on the numeric rating scale), located in the left upper and mid-abdomen, persistent in nature, with tenderness and rebound tenderness, accompanied by nausea and vomiting. There was no fever, jaundice, or diarrhea; stool and urine output were normal. His highest recorded serum triglyceride level during previous episodes was 30.35 mmol/L (reference range 0.48–2.3 mmol/L). Physical examination on admission revealed a height of 135 cm and weight of 30 kg, both below the 10th percentile for age based on Chinese reference growth curves (7). The patient had a strong dietary preference for soy products, with a marked aversion to carbohydrate-rich foods. There was no family history of pancreatitis or known metabolic disorders, although his mother and sister were noted to be obese.

### Laboratory tests and CT image

The patient presented with left upper quadrant abdominal pain. He had a history of multiple prior episodes of acute pancreatitis, raising the possibility of acute recurrent pancreatitis. However, based on the initial presentation alone, it was unclear whether this represented an acute episode or an acute exacerbation of chronic pancreatitis. Two laboratory evaluations were performed on the day of admission.

During the early morning emergency department visit, serum amylase was 51 U/L (reference range 30–110 U/L) and serum lipase was 216 U/L (reference range 23–300 U/L), both within normal limits; the initial sample was noted to be severely lipemic. No abnormalities were found in the abdominal ultrasound. Repeat testing approximately 6 h later revealed a serum amylase of 100 U/L and serum lipase of 560 U/L-the latter representing less than twice the upper limit of normal. Further evaluation revealed marked hypertriglyceridemia, with a serum triglyceride level of 28.96 mmol/L (12.6 times the upper limit of normal). This finding not only explained the observed severe lipemia but also suggested hypertriglyceridemia-induced acute pancreatitis (HTG-AP) as the most likely etiology. Notably, marked hypertriglyceridemia can interfere with enzymatic assays and may result in falsely normal or only mildly elevated pancreatic enzymes. Liver and kidney function parameters were within normal limits (Table 2).

**Table 2:** Changes in laboratory parameters across three hospitalizations.

Blood examination	Reference range	First *hospitalization*	Second *hospitalization*	Third *hospitalization*
Hemoglobin (g/L)	118–156	139-116-113*	129-130	145
Neutrophils (×10^9^/*L*)	1.6–7.8	9.9-9.7-1.6	5.2-1.9	7
Platelet (×10^9^/*L*)	100–300	273-208-221	207-261	240
Amylase (U/L)	30–110	51-100-68-87	65-54	47-69-63
Lipase (U/L)	23–300	216-560-81-58	187-22	63-82-66
Triglyceride (mmol/L)	0.48–2.3	28.96-2.6-15.63-4.25	45.57-30.85-14.19-14.66	74.68-3.84-3.77
Total cholesterol (mmol/L)	2.3–5.7	7.06-4.69-9.7-6.56	10.22-8.02-6.91-6.23	11-2.92-4.62
High density lipoprotein (mmol/L)	0.9–1.68	0.84-1.2-2.77-1.13	1.89-1.14-0.86-0.57	1.14-0.67-0.54
Low density lipoprotein (mmol/L)	1.4–3.7	3.57-2.23-4.47-3.69	5.07-3.59-3.6-3.27	5.86-1.19-2.96
Glucose (mmol/L)	3.9–6.1	6.07-8.43-2.98-3.94	6.07-4.38	4.59
Ammonia (*μ*mol/L)	10–47	40-42	42	34
Urea (mmol/L)	3.2–7.1	6.1-4.8-1.87-4.35	5.8-4.45	5.6
Creatinine (μmol/L)	58–110	40.1-23-28.1-49.6	33.8-35	36.6
Albumin (g/L)	40–55	41.1-41.3	42.9	38.3
Total bilirubin (µmol/L)	3–22	11.2-7.6	18.6	11.6
Alanine aminotransferase (U/L)	0–50	14–16	16	15
Aspartate aminotransferase (U/L)	17–59	28–30	31	23
Alkaline phosphatase (U/L)	45–125	203-121	ND**	ND
*γ*-Glutamyl transpeptidase (U/L)	10–60	12-12-13	10	13
Total bile acid (µmol/L)	0–15	0.43	ND	ND
C-reactive protein (mg/L)	0–5	10.1-158.25-4.46	25.81-3.43	1.86
Lactate (mmol/L)	0.5–1.6	1.7-0.9	ND	ND

* Hyphen-separated values indicate sequential results at different time points during each hospitalization.

** ND, not done.

**Figure 1:**
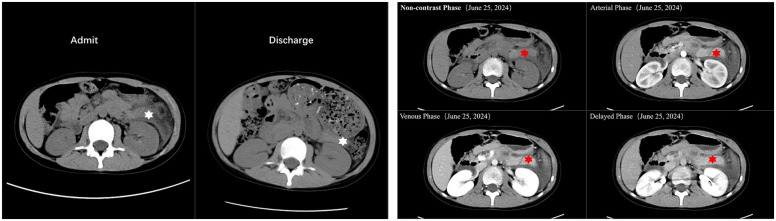
Contrast-enhanced abdominal CT scan of the patient confirmed acute pancreatitis. The pancreas (asterisk) enhances completely and was mildly enlarged without pancreatic duct dilatation or parenchymal necrosis. Marked inflammatory infiltrates and fluid collections are present around the pancreatic body and tail.

Because pancreatic enzyme levels were non-diagnostic, probably due to severe lipemia, and the diagnosis remained clinically uncertain, contrast-enhanced abdominal CT was performed. Contrast-enhanced abdominal CT demonstrated diffuse pancreatic enlargement with peripancreatic fat stranding, without evidence of chronic pancreatitis features such as pancreatic calcification, main pancreatic duct dilatation, or parenchymal atrophy. No pancreatic parenchymal necrosis, biliary obstruction, or pseudocyst formation was observed (Figure 1, Supplementary Video S1). Therefore, the patient met the diagnostic criteria for acute pancreatitis based on the combination of typical clinical presentation and characteristic CT findings, with no imaging evidence of chronic pancreatitis. The markedly elevated serum triglyceride level of 28.96 mmol/L (exceeding the threshold of 11.3 mmol/L for hypertriglyceridemia-induced pancreatitis) was identified as the most likely etiological factor.

Given the recurrent nature of pancreatitis, extreme hypertriglyceridemia, unusual dietary history, and absence of common causes (biliary, drug-induced, traumatic, infectious), a genetic etiology was suspected. Although Plasma amino acid and acylcarnitine profile analyses and whole-exome sequencing (WES) was suggested during the current hospitalization, the mother declined. However, she provided a WES report from a prior hospitalization several months earlier, which revealed compound heterozygous pathogenic variants in the *SLC25A13* gene: a paternal frameshift variant c.852_855delTATG (p.Met285Profs*2) in exon 9 and a maternal splicing variant c.615+5G>A(p.Ala206Valfs*7) in intron 6 (Supplementary Figure S1). These findings confirmed the diagnosis of citrin deficiency. Segregation analysis confirmed that both parents were heterozygous carriers of one mutation each, with no relevant medical history of metabolic or pancreatic diseases. Blood ammonia was normal (40 μmol/L; reference range 10–47 μmol/L).

### Amino acid and acylcarnitine profiles

Plasma amino acid and acylcarnitine profiling performed 7 days after discharge showed citrulline of 26.5 μmol/L (reference range 7.5–35 μmol/L) and arginine of 11.65 μmol/L (reference range 1–55 μmol/L), both within the reference range. Other amino acids were also unremarkable. The plasma ammonia level was within the reference range (<40 μmol/L), consistent with the compensated state characteristic of FTTDCD.

Given the unavailability of liver or pancreatic tissue biopsy in this case, immunohistochemical analyses could not be performed. However, based on the genetic confirmation and characteristic biochemical profile, the diagnosis of citrin deficiency was firmly established.

Regarding the absence of documented NICCD in early infancy for this patient, the patient's early medical records from birth are no longer traceable. However, upon specific questioning, the parents recalled that the patient had prolonged neonatal jaundice that resolved later than expected. Although we cannot retrospectively confirm a formal diagnosis of NICCD due to lack of contemporaneous laboratory data, this history raises the possibility that a mild or subclinical form of NICCD may have occurred during the neonatal period. Thus, the current presentation with FTTDCD may represent a continuum of the same disease rather than a complete absence of the NICCD stage.

### Acute management

Initial acute management included fasting, intravenous fluids, octreotide infusion to reduce pancreatic secretions, and a single session of plasma exchange, which rapidly lowered triglyceride levels to 5.2 mmol/L within 48 h. Upon genetic confirmation, dietary therapy was initiated following consultation with a metabolic dietitian: The recommended nutrient ratio is protein 15%–25%, fat 40%–50%, and carbohydrates 30%–40%, corresponding to an approximate ratio of 2:5:3 among the three nutrients. Specifically, the patient was advised to consume about 150 g of rice or other noodle-based carbohydrates, about 100 g of meat, and 400–500 mL of whole milk daily, along with appropriate supplementation of vegetables or legumes, adjusting daily dietary variety according to a nutritionist's recommended ratio. Additionally, MCT oil (5 mL per dose, three times daily with meals) and vitamin D (800 IU daily) were supplemented daily. Because plasma ammonia was normal, no additional sodium pyruvate or arginine was administered; however, an 18-amino acid intravenous formulation [Paediatric Compound Amino Acid Injection (18AA-I), containing 1.348 g of total amino acids per 20 mL] was administered at a daily dose of 9.36 g to meet metabolic demands. Symptoms resolved completely, and lipid profiles normalized by the time of discharge on day 14**.**

### Follow-up and clinical course

The patient was discharged on day 14 with strict dietary instructions. It is generally believed that the risk of HTG-AP recurrence rises with increasing triglyceride levels; thus, strict lipid monitoring is recommended for patients after discharge. During the first year of follow-up, he was readmitted twice with recurrent acute pancreatitis due to dietary non-adherence. In both incidents, he consumed high-carbohydrate meals (rice and sweets) against medical advice, causing his triglyceride levels to rise to 45.57 mmol/L and 74.68 mmol/L, respectively. Each incident required rehospitalization, temporary fasting followed by dietary adjustments, and the prescription of fibrate drugs to lower lipids. These events highlight the importance of strictly adhering to dietary guidelines in the management of CD.

Over the 20 months following diagnosis, the patient exhibited improved yet inconsistent adherence to the prescribed diet. Laboratory blood investigations demonstrated triglyceride concentrations ranging from 0.37 to 74.74 mmol/L (Supplementary Figure S2), whereas liver enzymes, glucose and ammonia levels remained within normal reference ranges. Abdominal ultrasound showed no evidence of hepatic steatosis. Modest improvements were observed in the patient's growth parameters (as of March 18, 2026, height: 158 cm, weight: 41 kg, corresponding approximately to the 40th and 30th percentiles, respectively.). No recurrent episodes of pancreatitis were documented during periods of reported dietary compliance.

## Discussion

Unlike previously reported CD-associated pancreatitis cases, which all occurred in the AACD stage with elevated ammonia and citrulline (5, 6), our patient was in the FTTDCD stage with normal ammonia and citrulline levels. Additionally, our patient presented with severe hypertriglyceridemia-a feature not documented in earlier reports (Table 1). This discrepancy suggests that pancreatitis can also occur earlier in the disease course, even without hyperammonemia, and that hypertriglyceridemia may be a key driver in such cases.

Delayed diagnosis in this patient (approximately two years from symptom onset) was multifactorial. His characteristic preference for high-fat foods was misinterpreted as simple picky eating, and recurrent abdominal pain was initially attributed to acute pancreatitis caused by overeating. Mild lipid abnormalities on routine testing were also easily attributed to dietary factors, whereas other typical abnormalities of CD may not manifest until the AACD stage, further hindering early recognition. Notably, a distinct dietary preference for protein- and fat-rich foods with carbohydrate aversion, accompanied by growth retardation, is highly suggestive of CD. Importantly, normal plasma citrulline and ammonia levels do not exclude FTTDCD, and a high index of clinical suspicion is essential.

If the patient's condition is not controlled and the inherent adaptive response persists, leading to a further increase in blood lipids, recurrent episodes are very likely to occur. In children with recurrent hypertriglyceridemic pancreatitis, the differential diagnosis includes primary familial lipid disorders, as well as secondary factors such as obesity, diabetes, and drug exposure (1). Notably, the combination of marked growth retardation, characteristic carbohydrate aversion, and absence of familial dyslipidemia renders primary lipid disorders less likely and instead raises suspicion for citrin deficiency.

We hypothesize that compensatory mechanisms inherent to CD itself contribute to elevated triglyceride levels, and the body's adaptive preference for a high-fat diet acts synergistically to further increase serum lipid levels, thereby triggering episodes of hypertriglyceridemic pancreatitis (1, 4). In CD patients, citrin deficiency im-pairs the function of the malate-aspartate NADH shuttle, resulting in an increased cytoplasmic NADH/NAD⁺ ratio and a decrease in cytoplasmic aspartate (2–4). To counterbalance this metabolic disturbance, the citrate-malate shuttle is enhanced, transferring acetyl-CoA from mitochondria to the cytoplasm to promote fatty acid synthesis (4); alternatively, enhancement of the glycerol phosphate shuttle may occur, but the key enzyme glycerol-3-phosphate dehydrogenase 2 is poorly ex-pressed in the liver, leading to accumulation of glycerol and glycerol-3-phosphate (4), further exacerbating hyperlipidemia. Additionally, by blocking the conversion of lactate to pyruvate and glycerol-3-phosphate to dihydroxyacetone phosphate, gluconeogenesis is impaired and blood lipids also increase (3). Consistent with this hypothesis, elevated levels of glycerol and glycerol-3-phosphate have been observed in the urine of FTTDCD patients (8). These mechanisms may underlie the pathogenesis of severe hyperlipidemia in our case.

The heterogeneity of CD-related complications across different reports may reflect distinct mechanisms and genetic heterogeneity, warranting further clinical characterization and mechanistic studies. The patient in this report carried compound heterozygous pathogenic variants in the SLC25A13 gene: a frameshift variant (c.852_855delTATG, p.Met285Profs*2) in exon 9 and a splicing variant (c.615+5G > A, p.Ala206Valfs*7) in intron 6. The c.852_855del variant is a founder mutation in East Asian populations, leading to a premature stop codon and complete loss of citrin function (9). The c.615+5G>A variant has been reported to cause aberrant splicing, resulting in reduced expression of functional citrin (9, 10). Whether variants at different loci that affect citrin expression are associated with the development of pancreatitis remains to be further investigated. Early genetic counseling may help prevent intergenerational transmission of these disorders.

Management of CD requires an individualized multimodal approach due to its significant genotype-phenotype heterogeneity (11, 12). In this case, plasma exchange during the acute pancreatitis episode rapidly lowered blood lipids and relieved abdominal pain. During two recurrences in the follow-up period, we attempted a strict dietary regimen after a brief fasting period, which also significantly reduced blood lipids. This suggests that although hyperlipidemia at this stage can trigger acute pancreatitis, lipid accumulation can be significantly reduced as the energy ratio improves.

The core of comprehensive long-term management is strict dietary adjustment (high protein, high fat, low carbohydrate), supplementation with medium-chain triglycerides (MCT), and support with fat-soluble vitamins (4, 11, 13, 14), aiming to delay natural progression to AACD.

For FTTDCD patients like the present case, significantly elevated serum lipids easily trigger recurrent pancreatitis, thus continuous monitoring of lipid levels is required. Long-term management, in addition to monitoring potential progression to AACD, also requires evaluation of intrahepatic lipid accumulation and pancreatic endocrine and exocrine function to prevent progression to chronic organ damage. According to general dyslipidemia guidelines (15), the use of fibrates or *ω*-3 fatty acid supplementation may be considered for lipid-lowering therapy. As discussed above, we hypothesize that hyperlipidemia in this setting may represent a compensatory metabolic adaptation (e.g., alternative energy storage or disposal of reducing equivalents), although it paradoxically increases the risk of pancreatitis. The optimal lipid control level therefore requires further investigation. If metabolic dysfunction-associated fatty liver disease (MAFLD) develops, management should follow MAFLD guidelines (16). For patients living in regions with diets primarily rich in carbohydrates, reinforced interventions through family education are necessary to avoid high carbohydrate intake that may trigger metabolic deterioration (e.g., rice, noodles, sugary drinks, and high-fructose foods) (3, 4, 11, 15).

Based on this case, we propose that long-term follow-up for pediatric CD patients should address multi-system complications. Monitoring should include: first, assessment of general status (height, weight, activity level, cognitive function, neuropsychiatric symptoms); second, laboratory tests (blood glucose, plasma proteins, liver and kidney function, lipid profile, blood ammonia, and plasma amino acid and acylcarnitine profiles); third, pancreatic function [fecal elastase-1, serum amylase/lipase, fasting glucose, HbA1c, and pancreatic secretory trypsin inhibitor (PSTI/SPINK1) where available] (17); and fourth, hepatic surveillance (liver ultrasound every 6–12 months, serum alpha-fetoprotein). If neuropsychiatric symptoms develop, cranial magnetic resonance imaging (MRI) and electroencephalography (EEG) should be performed.

## Conclusions

This case demonstrates that recurrent acute pancreatitis associated with severe hypertriglyceridemia may be one of the dominant presentations of pediatric FTTDCD, even in the absence of hyperammonemia or hypercitrullinemia. In children with recurrent hypertriglyceridemia-induced acute pancreatitis, citrin deficiency should be considered when characteristic dietary preferences and growth impairment are present. Early metabolic evaluation, genetic testing, individualized dietary therapy, and sustained family education are essential for preventing recurrence and improving long-term outcomes.

## Patient perspective

The patient's mother shared the following:

“ My child has disliked rice and noodles since he was very young. He especially loves soy products. We always thought this was just picky eating and never imagined it could be a disease. He had visited several local hospitals when he felt unwell, but I was disappointed with the diagnostic process, so we later transferred him to a provincial hospital in another province. At that hospital, doctors conducted various tests for genetic metabolic diseases and provided some symptomatic supportive treatment. However, because I had an extreme lack of trust in the domestic healthcare system at that time, we did not take the initial hospitalization test results seriously, nor did we follow up in a timely manner.

It wasn't until our child's second relapse, when he was hospitalized at a local hospital, that I still deeply distrusted the medical team there. At the same time, I was even more worried that my children might face discrimination because of a genetic disease, so I even told the medical team that the genetic test results from the other hospital showed everything was normal. Later, the doctors on the medical team patiently explained the pathogenesis and treatment principles of genetic metabolic diseases to me multiple times. Their professional attitude and patient explanations gradually helped me overcome many of my previous stereotypes and misconceptions. Ultimately, based on the genetic test report I eventually provided, the medical team was able to confirm my child's diagnosis-it turned out his body cannot process carbohydrates properly.

After the diagnosis, the medical team developed a detailed dietary guidance plan for our child. Adjusting his diet was very difficult at first. Our child couldn't understand why he couldn't eat rice, and it was also very hard for us to completely control what he ate at school. It wasn't until he experienced two relapses in the past year that he became scared himself. Now he actively refuses high-carbohydrate foods on his own. Although he doesn't like the taste of MCT oil, he can accept it when mixed into dishes. This disease has taught our entire family how to eat healthily.

Throughout our medical journey, we have been very satisfied with the professional care provided by the medical team. It is precisely because of this trust and understanding that I am willing to share our child's case with the world, hoping that it will help other patients recognize this disease earlier.” This English translation maintains the authentic voice and emotional tone of the patient's mother while accurately conveying all medical information and personal experiences shared in the original Chinese text.

## Data Availability

The data presented in this study are included in the article and Supplementary Material. The raw genetic sequencing data are not publicly available due to patient privacy and ethical restrictions, but may be made available from the corresponding author upon reasonable request and with appropriate institutional approval.

## Supplementary material

Supplementary Figure S1 The whole-exome sequencing results of the SLC25A13 gene in the proband and his family members. The whole-exome sequencing verification results of the SLC25A13 gene in the proband with citrin deficiency and his parents and elder sister. The proband carried compound heterozygous variants in SLC25A13: c.852_855delTATG and c.615+5G>A. The father carried c.852_855delTATG, whereas the mother and elder sister carried c.615+5G>A. Note: The variant sites are marked within the red boxes. The c.852_855delTATG variant represents a deletion of TATG at coding positions 852-855 in exon 9. The c.615+5G>A variant is a splice-site variant located at the fifth nucleotide of intron 6 and is shown as overlapping G/A peaks on sequencing.

Supplementary Figure S2 Serial triglyceride (TG) measurements in the patient during the follow-up. Triglyceride (TG) levels (mmol/L) during clinical follow-up. Data points (n = 12) were obtained from October 2024 to March 2026. The light blue-shaded area represents the normal reference range (0.48-2.3 mmol/L). A scale break is introduced on the y-axis to clearly demonstrate both extremely elevated TG levels (peak: 74.6 mmol/L) and near-normal values. Observed values are shown as black solid circles connected by a black line, with individual TG concentrations labeled above each point. Abbreviations: TG, triglyceride; This figure was generated by R4.5.0.
