# Supplementary figures and images for: A pediatric case of citrin deficiency presenting with recurrent hypertriglyceridemic pancreatitis-a case report
Source: Front Pediatr. 2026 Jun 8;14:1816553. doi: 10.3389/fped.2026.1816553 (PMC13283877; doi:10.3389/fped.2026.1816553)

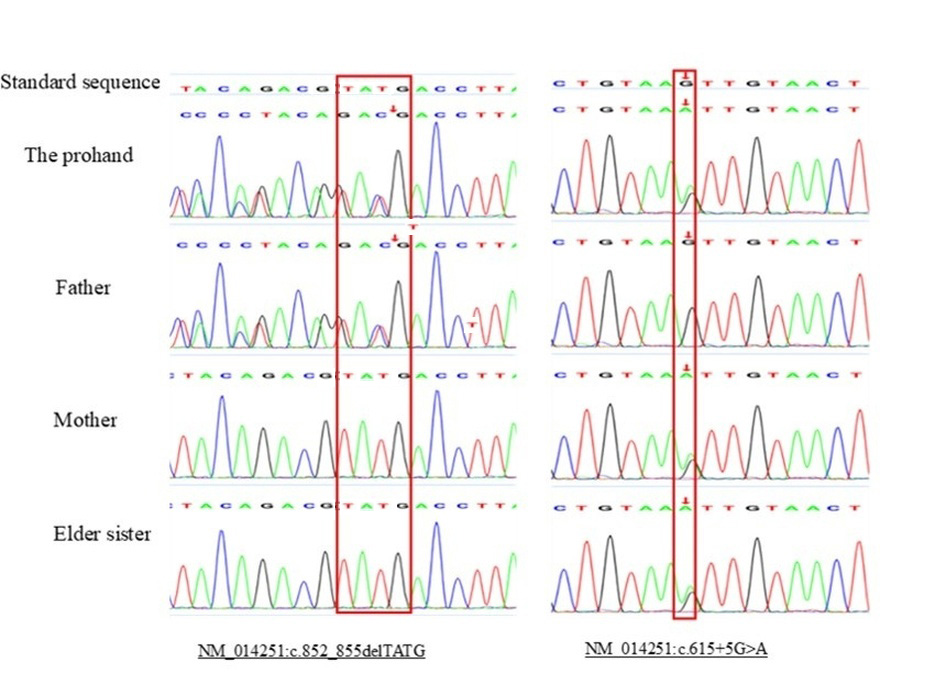

Supplement: Supplementary Figure S1 — The whole-exome sequencing results of the SLC25A13 gene in the proband and his family members. The whole-exome sequencing verification results of the SLC25A13 gene in the proband with citrin deficiency and his parents and elder sister. The proband carried compound heterozygous variants in SLC25A13: c.852_855delTATG and c.615+5G>A. The father carried c.852_855delTATG, whereas the mother and elder sister carried c.615+5G>A. Note: The variant sites are marked within the red boxes. The c.852_855delTATG variant represents a deletion of TATG at coding positions 852-855 in exon 9. The c.615+5G>A variant is a splice-site variant located at the fifth nucleotide of intron 6 and is shown as overlapping G/A peaks on sequencing. [file Image1.jpg]

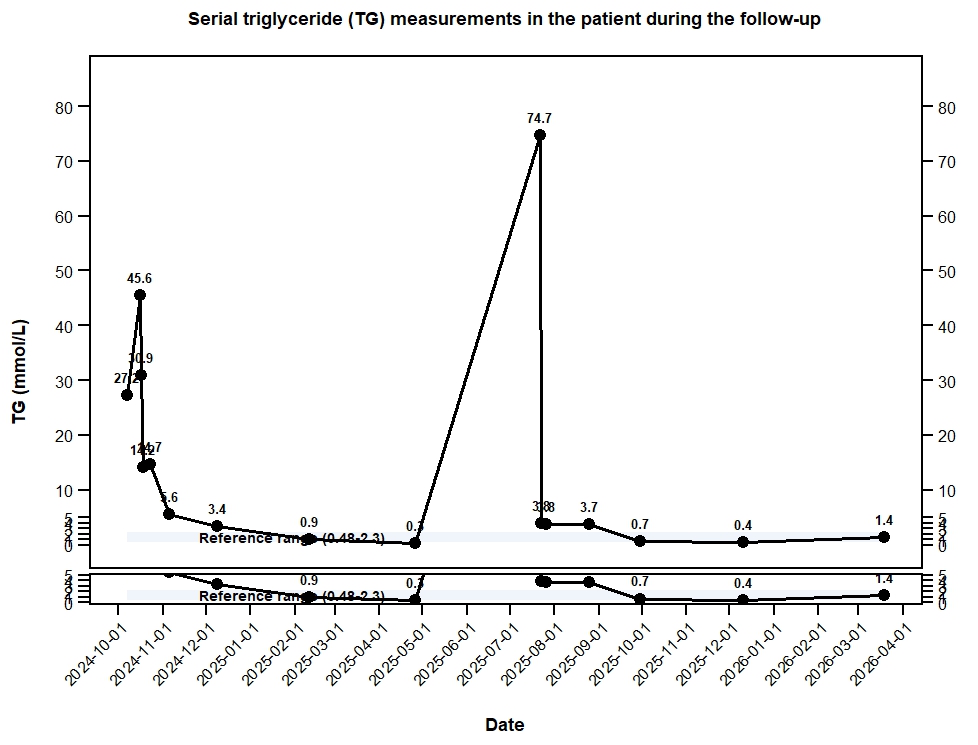

Supplement: Supplementary Figure S2 — Serial triglyceride (TG) measurements in the patient during the follow-up. Triglyceride (TG) levels (mmol/L) during clinical follow-up. Data points (n = 12) were obtained from October 2024 to March 2026. The light blue-shaded area represents the normal reference range (0.48-2.3 mmol/L). A scale break is introduced on the y-axis to clearly demonstrate both extremely elevated TG levels (peak: 74.6 mmol/L) and near-normal values. Observed values are shown as black solid circles connected by a black line, with individual TG concentrations labeled above each point. Abbreviations: TG, triglyceride; This figure was generated by R4.5.0. [file Image2.jpeg]
